# Nicotine enhances the ability of cues to control behavior and evoke dopamine release in the dorsolateral striatum

**DOI:** 10.1016/j.jpet.2025.103662

**Published:** 2025-07-22

**Authors:** Michael Z. Leonard, Hannah B. Elam, Hye Jean Yoon, Sofia H. Lago, Megan E. Altemus, Shemuel Roberts, Maxime Chevée, Erin S. Calipari

**Affiliations:** 1 Department of Pharmacology, Vanderbilt University, Nashville, Tennessee; 2 Vanderbilt Center for Addiction Research, Vanderbilt University, Nashville, Tennessee

**Keywords:** Conditioned reinforcement, Cue, Reinforcer, Second order schedule

## Abstract

Nicotine is one of the most widely used addictive substances, yet its primary reinforcing effects are relatively weak. Nicotine’s ability to potentiate responding for conditioned reinforcers is thought to drive persistent drug use. Here, we show that nicotine failed to alter responding maintained by a primary sucrose reinforcer (under a variable-ratio [VR] 11 schedule) across a broad dose range (0.01–1.0 mg/kg). Yet, nicotine enhanced operant behavior under a second-order reinforcement schedule in which responses produced sucrose-associated cues and were only intermittently reinforced by sucrose itself. Together, we demonstrate that nicotine selectively augments behavior maintained by conditioned reinforcers (sucrose-associated cues) in a rate-dependent manner, increasing responding only in mice with low rates of reinforcement behavior at baseline. Using fiber photometry, we demonstrate that nicotine selectively amplified cue-evoked dopamine release in the dorsolateral striatum—but only in low baseline responders—while having no effect on dopamine signaling in the nucleus accumbens. These effects were blocked by the nicotinic receptor antagonist mecamylamine. Further, nicotine’s influence on behavior was abolished when the contingency between action and conditioned stimuli was disrupted, indicating that nicotine strengthens cue control of behavior rather than increasing motivation generally. Collectively, these findings reveal that nicotine’s behavioral actions emerge through an interaction between pharmacological mechanisms, behavioral contingencies, and individual differences in baseline behavioral control.

**Significance Statement:**

Nicotine strengthens the impact of environmental cues on behavior by amplifying dopamine signals in specific projection targets, but only in individuals with specific behavioral traits. This reveals how nicotine hijacks learning processes to promote persistent, cue-driven actions.

## 1 Introduction

Nicotine is a highly abused substance among humans, yet its primary reinforcing effects are paradoxically weak. Indeed, humans self-report lower positive subjective feelings for nicotine as compared to a wide range of other drugs of abuse.^1^ Accumulating evidence indicates that nicotine’s ability to enhance the reinforcing value of associated cues plays a crucial role in maintaining smoking behavior as its effects are limited in the absence of these conditioned cues.^2^, ^3^, ^4^ In fact, humans will continue to smoke denicotinized cigarettes at similar levels in laboratory settings, presumably maintained by the sensory stimuli involved in smoking.^5^, ^6^, ^7^ Similarly, environmental cues associated with nicotine delivery are capable of maintaining nicotine-seeking behavior in animal models, even in the absence of the drug’s pharmacological effects.^8^, ^9^, ^10^, ^11^, ^12^ Thus, the effects of nicotine cannot simply be explained by the pharmacological actions of the drug at its target, but rather arises from its ability to modulate how cues influence behavior.

Importantly, nicotine’s effects on cue-mediated behavior extend beyond the drug context. For example, nicotine increases responding for presentation of sensory stimuli (eg, light cues and tones^13^, ^14^, ^15^, ^16^, ^17^) as well as cues associated with nonpharmacological reinforcers such as food.^18^, ^19^, ^20^, ^21^ Together, interactions between the pharmacological effects of drugs and nonpharmacological environmental stimuli are critical for determining the effects of nicotine on behavior. An important question remains: are the mechanisms controlling nicotine’s effects on behavior different when behavior is maintained by a primary reinforcer as compared to conditioned cues?

Both the primary reinforcing effects of nicotine, as well as its ability to enhance the reinforcing properties of cues, are dependent on nicotine actions on dopamine systems. Direct nicotinic-receptor-mediated actions on dopamine neurons are essential for both nicotine self-administration^22^, ^23^, ^24^, ^25^, ^26^, ^27^, ^28^ as well as the ability of nicotine-predictive cues to support reinforcement (ie, conditioned reinforcement^17^^,^^29^). Nicotinic receptors are located both on dopamine neurons in the midbrain and at distal axon projections throughout the reward circuitry.^30^ Critically, effects on these systems in different dopamine projection targets throughout the brain are known to control disparate aspects of reinforcement. For example, primary reinforcement is highly dependent on increases in dopamine release in ventral regions of the striatum,^31^, ^32^, ^33^ while conditioned responses to cues that promote habitual behavior are thought to depend on the dorsolateral striatum (DLS).^34^, ^35^, ^36^, ^37^ Thus, the major overarching question is whether the same drug differentially engages reinforcement circuits in the brain under conditions in which behavior is maintained by a primary vs a secondary (ie, cues) schedule of reinforcement.

Here, we combine operant behavior maintained by either a primary reinforcer (sucrose) or a secondary reinforcer (sucrose-conditioned cues) to understand the effect of nicotine on behavior under these conditions. By integrating these approaches with optical imaging techniques to record dopamine release in awake and behaving animals across multiple dopaminergic projection targets, we assessed the effects of nicotine on cue-evoked dopamine release in the dorsolateral versus ventral striatum. We find that nicotine’s effects are dependent both on baseline rates of behavior as well as the presence of conditioned cues. Nicotine’s actions emerge through an interaction between pharmacological mechanisms, behavioral contingencies, and individual differences at baseline.

## 2 Materials and methods

### 2.1 Animals

Eight-week-old male and female C57BL/6J mice (*n* = 26) were acquired from Jackson Laboratory. All mice were group-housed (5/cage) in a temperature-controlled vivarium with ad libitum access to food and water. Lights were maintained on a 12:12 hour reverse light/dark cycle (lights off at 08:00), where testing was conducted during the dark period. All procedures were conducted in accordance with National Institutes of Health guidelines for animal care and use^38^ and were approved by the Institutional Animal Care and Use Committee of Vanderbilt University Medical Center.

### 2.2 Drugs

Nicotine hydrogen tartrate salt (Sigma Aldrich) was dissolved in sterile 0.9% saline, and the pH was adjusted to ∼7.2 with diluted NaOH. Acute nicotine doses (0.01, 0.03, 0.1, 0.3, 1.0 mg/kg, i.p.) were administered 10 minutes prior to testing. Pretreatment with mecamylamine hydrochloride (1 mg/kg, i.p.; Sigma Aldrich) was delivered 10 minutes before nicotine dosing. All drugs were administered at a volume of 1 mL/kg.

### 2.3 Apparatus

All experiments were conducted in standard-wide mouse operant conditioning chambers housed within sound-attenuating enclosures and operated by Med-PC software (Med Associates). Each chamber was equipped with 2 retractable levers with cue lights above them. Between the levers, a 3D-printed modular insert housed a stainless-steel cannula (lick port, 18 gauge) that was connected to a syringe pump for fluid delivery and attached to a contact lickometer (Med Associates) for lick detection. A house light was located opposite the levers and lick port.

### 2.4 Behavioral procedure

#### 2.4.1 First-order schedule: VR11

Mice were initially trained to respond for 20% sucrose under fixed-ratio 1 (FR1) reinforcement schedule during 1-hour daily sessions. Responses on the active lever extinguished the house light and produced sucrose delivery (10 *μ*L), paired with a 3-second audiovisual stimulus (tone + cue light; conditioned stimulus [CS]). Responses on the inactive lever were recorded but had no scheduled consequences. Mice were progressively transitioned to a first-order, variable-ratio (VR11) schedule that consisted of 4 potential ratio requirements: FR1, FR3, FR10, and FR30. After 3 consecutive sessions with stable response rates (±20%), nicotine doses were administered in a counterbalanced order across animals. At least 2 training sessions were conducted between tests to ensure recovery to predrug response levels.

#### 2.4.2 Second-order schedule: FR4(VR11:CS+)

**Fig. 1:**
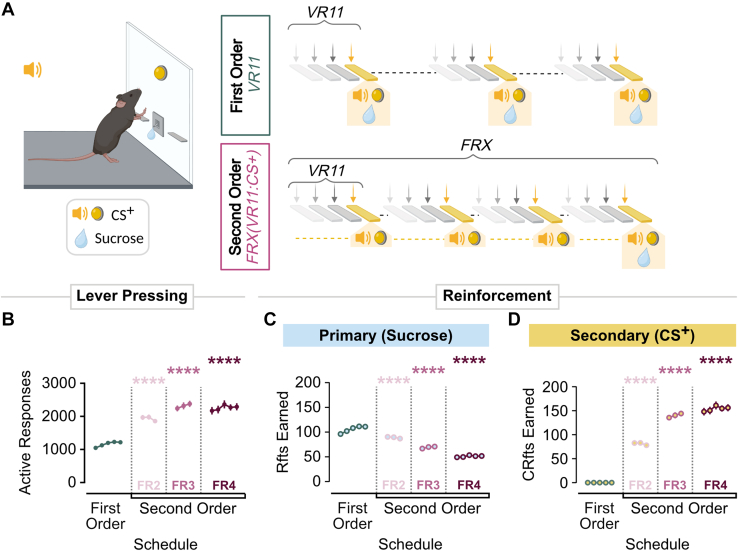
Second-order schedules maintain high response rates despite infrequent sucrose delivery. (A) Schematic of first-order and second-order operant procedures. First order: behavior was maintained on a variable-ratio 11 (VR11) schedule, in which completion of each unit schedule resulted in the delivery of sucrose. Second order: behavior was maintained on a VR11 schedule, in which completion of the unit schedule resulted in the delivery of a conditioned stimulus (VR11:CS+), without sucrose. Under the terminal schedule, every fourth unit completion resulted in concurrent conditioned stimulus + sucrose delivery. (B) Total lever presses emitted at each phase of training. Animals were initially trained under a first-order schedule (VR11; green) in which sucrose was delivered following every unit completion. Mice were then moved to a second-order schedule in which sucrose was delivered less frequently: every other unit completion (FR2; light pink), every third (FR3; pink), and every fourth (FR4; dark pink). (C) Number of primary reinforcers earned per session decreased while the (D) CS+ presentations earned increased over schedule presentations (under VR11 from FR2–FR4). Data are depicted as mean ± SEM. ∗∗*P* < .01, ∗∗∗*P* < .001, ∗∗∗∗*P* < .0001.

Upon completion of testing under a first-order VR11, mice were trained to respond under a second-order schedule for sucrose. Second-order schedules have been widely used in the analysis of operant behavior as a tool for examining how environmental stimuli associated with reinforcement come to exert control over instrumental behavior.^39^, ^40^, ^41^ Throughout training, sucrose-paired cues (CS+) acquire motivational value through their conditioned association, such that brief presentations of these cues in the absence of sucrose are sufficient to enhance and sustain operant responding over long interreward periods.^42^^,^^43^ Here, mice responded under a VR11 schedule (ie, the unit schedule; VR:CS+) for a brief presentation (1 second) of the audiovisual cue associated with sucrose delivery (CS+). Responding on this unit schedule was then reinforced by sucrose according to a FR4 schedule of reinforcement, such that every fourth completion of the unit schedule resulted in sucrose delivery (see Fig. 1). As in the first-order VR11 schedule, the unit schedule comprised 4 components (FR1, FR3, FR10, and FR30) that were presented in a random order. The number of response units required per sucrose reinforcer was incrementally increased across training, with each transition phase comprising 3 sessions. Nicotine testing began after ≥5 sessions on the terminal second-order schedule, FR4(VR11:CS+).

#### 2.4.3 Cue contingency degradation: FR4(VR11:x)

When conditioned stimuli exert control over operant behavior, unpairing these cues from the actions that produce them—or removing them entirely—consistently results in reduced responding.^39^^,^^42^, ^43^, ^44^ Therefore, a contingency degradation procedure was used to establish the extent to which CS+ presentations influenced response rates under the second-order schedule. After drug testing was complete, mice were given 4 additional sessions under the second-order schedule to re-establish stable response rates. Subsequently, 5 contingency degradation sessions were conducted, wherein CS+ presentations were uncoupled from the completion of the unit schedule. While the total number of lever responses required per sucrose delivery (FR44) was held constant, cue presentations were instead delivered on a variable time schedule (VT20) that approximated the average CS+ presentation rate observed under the second-order schedule. This manipulation aimed to maintain primary reinforcement rate, while removing the contingency between lever responses and cue presentation (ie, conditioned reinforcement).

After 5 sessions of contingency degradation, mice were given one additional nicotine test (0.1 mg/kg) under the uncoupled-CS+ procedure to determine whether nicotine’s behavioral effects require the availability of conditioned reinforcers.

### 2.5 Fiber photometry

#### 2.5.1 Surgery

To measure dopamine release in the striatum, a subset of mice (*n* = 10) was bilaterally injected with a virus encoding the dopamine sensor dLight1.1 (AAV5.CAG.dLight1.1, Addgene) into the nucleus accumbens (NAc) core and the DLS. Stereotaxic surgery was performed under isoflurane anesthesia. The virus was delivered using a 10-*μ*L NanoFil syringe (World Precision Instruments) at a rate of 100 nL/min to a total of 500 *μ*L per site. Injection coordinates were calculated from a stereotaxic atlas^45^ to target the right NAc: anteroposterior: +1.5 mm, mediolateral: +1.5 mm with a 10° angle from midline, dorsoventral: −4.4 mm from skull surface; or the left DLS: anteroposterior: +0.5 mm, mediolateral: −2.5 mm, dorsoventral: −3.1 mm. The needle was left in place for 7 minutes before being slowly withdrawn from the injection site. Fiber optic ferrules (400 *μ*m, 0.48 numerical aperture; Doric Lenses) were subsequently implanted 0.1 mm above either target region and affixed to the skull with Metabond (Parkell) and carbon-pigmented dental cement (Lang Dental). Ketoprofen (5 mg/kg, s.c.) was administered prior to surgery and for ≥3 days postsurgery for analgesia. Animals were returned to group housing immediately after surgery and allowed to recover for a minimum of 3 weeks to allow for optimal viral expression before commencing experiments.

#### 2.5.2 Data acquisition

Fiber photometry was conducted using an RZP5 data acquisition system with an RZ10X (Tucker-Davis Technologies) coupled to a 3-m optic fiber cable (Doric Lenses). Two light-emitting diodes (Thorlabs) controlled by an LED driver (Thorlabs) emitted 470 nm and 405 nm (isosbestic control) light at 50 *μ*W or 30 *μ*W, respectively, at the tip of the optic fiber. Fluorescent emissions were collected by a photoreceiver (Newport Visible Femtowatt Photoreceiver Module; Doric Lenses), at 1017-Hz sampling rate. The onset of key behavioral events (eg, CS+ onset, lever press, spout lick) were communicated via transistor–transistor logic signals, routed from the Med-PC control interface (Med Associates) directly into the digital input port of the RZP5. The acquisition of photometry signals and behavioral transistor–transistor logics was synchronized using Synapse software (Tucker-Davis Technologies), which simultaneously controlled LED intensity and timing via the RZP5.

#### 2.5.3 Data processing

Processing and analysis of fiber photometry data was conducted using a custom Python pipeline. Raw photometry data was preprocessed according to parameters described by Simpson et al.^46^ Briefly, a 10 Hz low-pass filter was applied to the 470 nm and 405 nm signals to reduce nonphysiological, high-frequency noise using the Scipy function *filtfilt*. Filtered signals were then fitted with a double exponential curve to estimate the time course of photobleaching, which was corrected by subtracting the fit from either signal. Next, motion artifacts were estimated from the 405 nm isosbestic channel via linear regression, whereby coincident signal fluctuations between the 470 nm and 405 nm signal were attributed to movement. Finally, the movement-corrected signal was normalized to basal fluorescence to produce a dF/F trace. All traces were down-sampled to 20 Hz for analysis.

#### 2.5.4 Peri-event analysis

Dopamine fluctuations around behavioral and stimulus events were quantified and compared across mice by transforming each dF/F trace into trial-wise *z*-scored values. *Z*-scores were calculated using the mean and SD from a 3-second period immediately preceding the event of interest. However, as sucrose collection was typically preceded by CS+ presentation, dLight responses to sucrose licks were standardized to the pre-CS+ baseline. The primary dependent measure for peri-event dLight responses was peak height, which was identified for each trace as the maximum absolute *z*-score value observed within a 2-second window after an event. Although all events were analyzed across each recording session, only the first 4 trials were considered for analysis (ie, 12 CS+ presentations, 4 sucrose presentations) to permit comparisons across all mice under all conditions.

### 2.6 Histology

Upon conclusion of experiments, mice were transcardially perfused with PBS and 4% paraformaldehyde under ketamine anesthesia (100 mg/kg, i.p.). Brains were extracted, fixed overnight in paraformaldehyde, and stored in 30% sucrose solution. Tissue was later sectioned into 40 *μ*m slices using a microtome and stained using an anti-GFP antibody (Aves Labs; 1:1000) targeting the viral-mediated dLight. After 24 hours, sections were transferred to AlexaFluor 488-conjugated secondary antibody solution (Jackson ImmunoResearch; 1:500) for 12 hours, and mounted. Images were used to confirm placement of optical fibers and dLight expression within the NAc and DLS and were acquired using a Keyence BZ-X700 inverted fluorescence microscope.

### 2.7 Statistical analysis

GraphPad Prism (v10.2) was used to statistically analyze and graph data. Drug effects on operant performance are expressed as a change in response rate from an established baseline under drug-free conditions. Baseline rates were initially determined for each animal as an average of the last 3 sessions prior to drug testing. Throughout testing, training sessions immediately preceding a drug test contributed to a 3-session rolling average against which drug-induced response rates were compared. Phenotypic subgroups were identified at the conclusion of drug testing and reflect an average of all baseline determinations collected throughout the second-order reinforcement studies (*n* = 10 sessions)

Behavioral variables were primarily compared using one- or two-way ANOVA. Main effects were followed up with Bonferroni multiple comparison tests. The relationship between baseline response rates and drug effects were evaluated by simple linear regressions applied to each independent dose. Nested analyses were used to compare peri-event dopamine responses, in which observations were nested by subject. Comparisons across multiple doses were achieved using nested ANOVA, while comparisons between 2 groups were conducted via nested *t* test.

## 3 Results

### 3.1 Rate-dependent effects of nicotine on operant behavior

Mice were trained on a second-order schedule of sucrose reinforcement to distinguish how reward-associated stimuli gain influence over operant behavior. Initially, mice performed on a first-order schedule, where sucrose was delivered at each completion of a VR11 schedule (Fig. 1A, top). With experience, cues that consistently accompany sucrose delivery (CS+) tend to acquire motivational significance through these conditioned associations and can maintain operant behavior even when presented without sucrose. Therefore, the presentation of the CS+ alone may sustain high rates of operant behavior. With implementation of the second-order schedule, completion of VR11 unit schedules resulted a brief presentation of the CS+, alone (VR11:CS+), and sucrose was only delivered after a fixed number (X) of unit schedules were completed (FRX[VR11:CS+]; Fig. 1A, bottom). The number of unit schedules required to obtain each sucrose reinforcer gradually increased to 4 over the course of training.

Over training, mice exhibited elevated levels of active lever pressing under second-order schedules, despite a marked reduction in sucrose deliveries as the reinforcement schedule became more stringent (Fig. 1, B and C). In the second-order conditions, as the response requirement was increased over training, presentation of the CS+ maintained response rates (Fig. 1D). Compared with the VR11 first-order schedule alone, a main effect of schedule on active lever responses was observed (*F*_3,204_ = 188.5, *P* < .0001), with increases at each second-order schedule: FR2 (Bonferroni *t* = 13.23, *P* < .0001), FR3 (Bonferroni *t* = 20.21, *P* < .0001), and FR4 (Bonferroni *t* = 20.52, *P* < .0001). Similarly, a main effect of schedule on sucrose reinforcers earned was observed (Fig. 1C; *F*_3,204_ = 53.47, *P* < .0001), with fewer reinforcers obtained at more demanding schedules (*P* < .0001 for all post hoc comparisons).

**Fig. 2:**
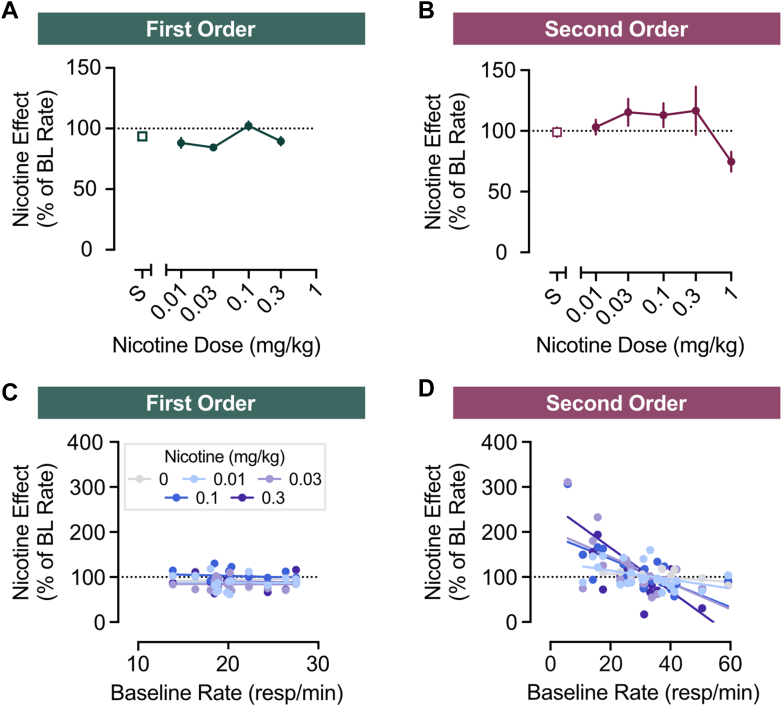
Effects of nicotine on behavior are schedule- and rate-dependent. Dose-response curves show no effects of nicotine on operant response rates relative to a drug-free baseline (BL) under either (A) a first-order VR11 schedule or (B) a second-order schedule. (C) The relationship between nicotine-elicited changes in response rate (resp/min; *y*-axis) versus baseline rate of behavior (*x*-axis). There is no relationship when behavior is maintained by a primary reinforcer. (D) Under a second-order schedule—in the same animals—there is a negative relationship between baseline response rates (*x*-axis) and drug-elicited changes in responding (*y*-axis). Data are presented as mean ± SEM.

Across a range of doses, nicotine did not consistently modulate behavior under either the first-order VR11 or the second-order schedule (Fig. 2, A and B). Under the first-order VR11 schedule, there was a modest main effect of nicotine treatment relative to baseline performance (*F*_4,60_ = 3.629, *P* < .05), but no single dose differed significantly from saline. Under the second-order schedule, no main effect of nicotine dose was detected (*F*_5,106_ = 1.416), although a planned comparison revealed that 1.0 mg/kg nicotine significantly disrupted responding relative to saline (Bonferroni-corrected *t* = 3.337, *P* < .05).

Although there were limited effects of nicotine at the group level, we observed notable variability across animals’ responding under a second-order schedule. It is well established that behavioral and subjective responses to nicotine can be heterogenous^47^ and may critically depend on the baseline rate of an observed behavior.^48,49^ Therefore, nicotine doses were plotted for each animal as a function of their baseline response rates under either a simple VR first-order schedule (Fig. 2C) or second-order schedule (Fig. 2D). Simple linear regressions were conducted to assess the relationship between baseline response rate and the change in response rate induced by nicotine doses. The behavioral effects of nicotine could be described as rate-dependent on responding under a second-order schedule. Linear regressions showed no relationship between baseline response rates and nicotine-induced changes in rates under the VR11 first-order schedule (*R*^2^ > 0.025 at all doses; see Supplemental Fig. 1). However, under the second-order schedule, nicotine dose-dependently modulated responding according to the animal’s baseline rate, and this relationship was most pronounced at the 0.03 (*R*^2^ = 0.398), 0.1 (*R*^2^ = 0.429), and 0.3 mg/kg (*R*^2^ = 0.345) doses (*P* < .01; see Supplemental Fig. 2).

**Fig. 3:**
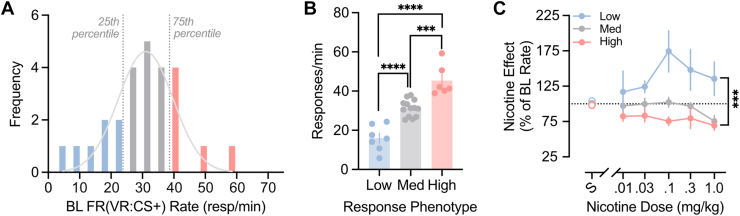
Baseline rate of responding predicts behavioral sensitivity to nicotine. (A) Frequency distribution of baseline (BL) response rates under the second-order schedule (mean = 30.12 responses/min; SD = 12.14). Dotted gray lines delineate the upper and lower 25% of response rates. (B) Baseline response rates differed significantly across subgroups, as expected. (C) Nicotine dose-response functions for second-order responding for sucrose, for Low (blue), Medium (gray), and High (red) baseline response-rate subgroups. Response rates were dose-dependently elevated in the low baseline responders. Data are presented as mean ± SEM. ∗∗*P* < .01, ∗∗∗*P* < .001, ∗∗∗∗*P* < .0001.

To further characterize behavioral performance as a predictor for both drug effects and underlying dopamine dynamics, mice were classified according to baseline response rates under the second-order schedule (Fig. 3A). Across all mice, basal response rates were normally distributed (mean = 30.12 responses/min, SD = 12.14; Gaussian fit *R*^2^ = 0.8084). Using a quartile split, mice were identified as either Low (>25^th^ percentile), Med (25^th^–75^th^ percentile) or High (>75^th^ percentile) rate phenotypes. One-way ANOVA confirmed that these groups differed significantly in their baseline response rates (Fig. 3B; main effects: *F*_2,23_ = 40.67, *P* < .0001; Bonferroni comparisons between each group: *P* < .001).

Response-rate phenotype was a clear determinant of nicotine’s behavioral effects across doses (Fig. 3C; main effects: *F*_2,25_ = 10.03, *P* < .001). The low baseline responders exhibited greater sensitivity to nicotine than both medium (Bonferroni *t* = 3.746, *P* < .01) and high-baseline responders (Bonferroni *t* = 4.197, *P* < .001). Neither the medium nor the high-baseline responders exhibited significant modulation of behavior by nicotine (main effects; Med: *F*_5,60_= 2.091, n.s.; High: *F*_5,25_= 2.091, n.s.), and no differences were observed between the groups (Bonferroni *t* = 0.9852, n.s.).

### 3.2 Nicotine enhances CS+-evoked dopamine release in the DLS

**Fig. 4:**
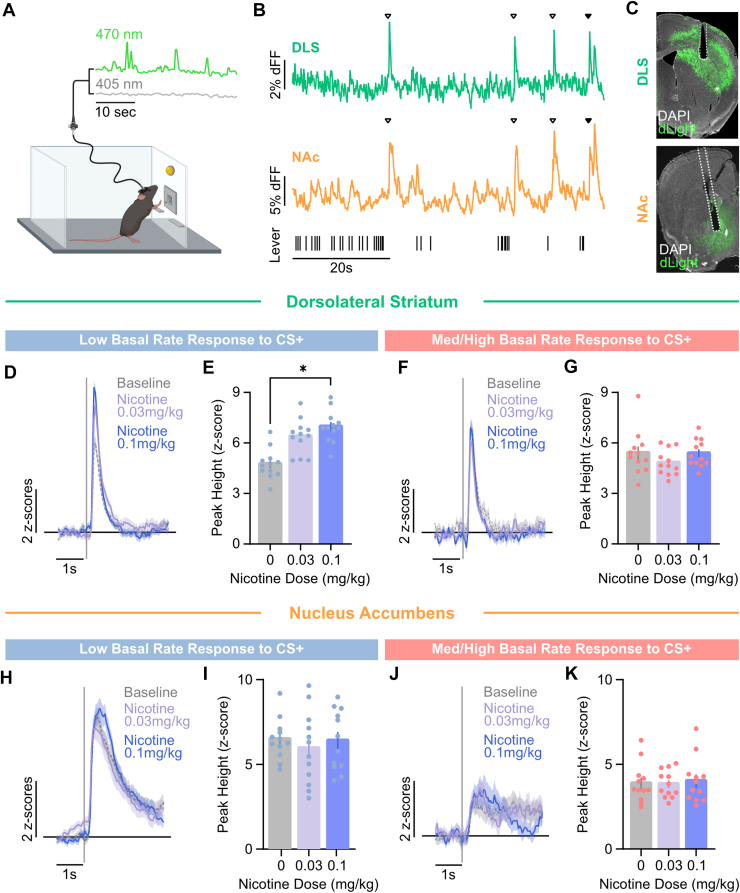
Nicotine differentially modulates dopamine responses to conditioned stimuli in the DLS in a response-rate dependent manner. (A) Schematic of fiber photometry recordings of dLight1.1 expressed in the DLS and NAc core of mice. (B) Representative photometry traces from the DLS (top) and NAc (bottom) of a single mouse. Arrows above the traces denote presentation of conditioned stimulus (CS+) either alone (open triangles) or with sucrose (closed triangle). Tick marks represent active lever presses. (C) Representative histology confirms dLight1.1 expression, showing the fiberoptic tract above the DLS (top) and NAc (bottom). dLight photometry traces from the DLS time-locked to CS+ (gray line) presentation in (D) low baseline and (F) medium and high-baseline responders. (E) In the DLS, nicotine dose-dependently enhanced peak CS+-evoked dopamine responses in mice with low baseline response rates but not (F) mice with medium and high-baseline response rates. dLight photometry traces from the NAc time-locked to CS+ presentation in (H) low baseline and (J) med/high-baseline responders. In the NAc, nicotine did not alter peak CS+-evoked dopamine responses in either (I) low or (K) med/high-baseline responders. DAPI, 4′,6-diamidino-2-phenylindole. Data are presented as mean ± SEM. ∗*P* < .05.

Dopamine transients were observed in both DLS and NAc at the onset of conditioned reinforcers (CS+) during behavior maintained by a second-order schedule of reinforcement (Fig. 4B). Because medium and high-baseline responders showed similar behavioral profiles in response to nicotine, these groups were combined (Med/High) for subsequent photometry experiments.

Within the DLS, CS+-evoked dopamine did not differ between subgroups during baseline task performance (Supplemental Fig. 3, A and B; *P* = .198). However, nicotine dose-dependently enhanced dLight signal around CS+ events in low baseline responders (Fig. 4D). A nested ANOVA revealed a main effect of dose (Fig. 4E; *F*_2,11_ = 4.284, *P* < .05) on peak dLight responses in the low baseline responders. Whereas these effects were modest at the 0.03 mg/kg dose (Bonferroni *t* = 1.954, n.s.), 0.1 mg/kg nicotine enhanced the peak dopamine release, compared with saline (Bonferroni *t* = 2.862, *P* < .05). Importantly, these effects were specific to the low baseline responders, as nicotine failed to modulate CS+-elicited dopamine in DLS of Med/High responders (Fig. 4, F and G; *F*_2,6_ = 1.630, n.s.).

In contrast, no effects of nicotine on CS+-evoked dopamine responses were observed in the NAc (Fig. 4, H–K; *F*_2,9_ = 0.1165, *P* = .8914), although low baseline responders exhibited elevated dopamine responses overall in the NAc (Supplemental Fig. 3, C and D; *F*_1,7_ = 13.77, *P* < .01).

### 3.3 Nicotinic receptor mediates rate-dependent effects of nicotine

**Fig. 5:**
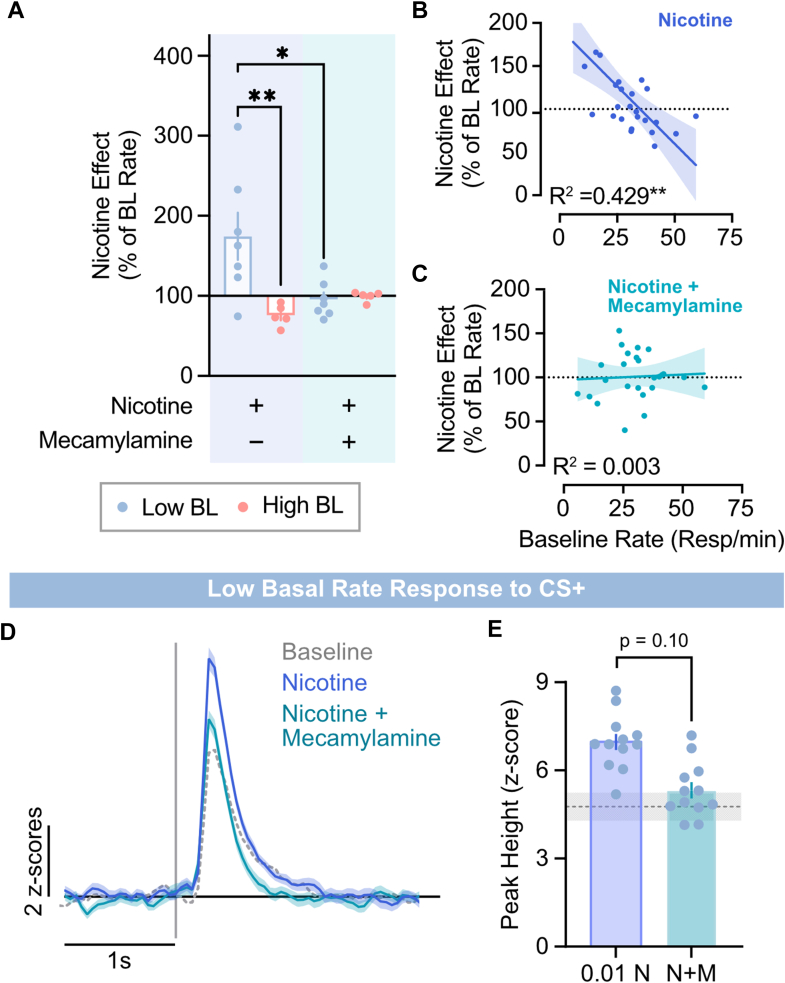
Nicotine’s rate-dependent effects on behavior and neural responses are reversed by mecamylamine. (A) Mecamylamine selectively decreased response-rates in the low baseline (BL) responders but had no effect on response rate in the high-baseline responders. (B) Relationship between baseline response rate (resp/min) and nicotine-induced responding. (C) Mecamylamine pretreatment eliminated the rate-dependent effects of nicotine. (D) dLight1.1 photometry traces from the DLS in response to CS+. (E) CS+-evoked dopamine responses were not reduced in the DLS after nicotine (N; 0.1 mg/kg, i.p.) and mecamylamine (M; 1 mg/kg, i.p.) administration compared with nicotine alone. Dotted gray line represents mean peak height at baseline, with shaded region denoting SEM. All data are presented as mean ± SEM.

Mecamylamine, a selective nicotinic acetylcholine receptor antagonist, reversed nicotine’s rate-dependent effects on response rate (Fig. 5). Two-way ANOVA revealed a main effect of the drug on response rates (Fig. 5A; *F*_3,20_ = 6.013, *P* < .01). Specifically, in low baseline responders, mecamylamine significantly attenuated the increase in response rates induced by 0.1 mg/kg nicotine (Bonferroni *t* = 3.288, *P* < .05). Linear regression also revealed that mecamylamine pretreatment was sufficient to abolish the relationships between baseline rates and nicotine-induced response changes (Fig. 5, B and C; *R*^2^ = 0.003).

Similarly, mecamylamine largely attenuated the capacity for nicotine to attenuate CS+-elicited dopamine release in the DLS, although this effect did not reach statistical significance (Fig. 5, D and E; *F*_1,6_ = 3.709; *t*_6_ = 1.926; *P* = .102). Mecamylamine alone had no effects on behavior or CS+-evoked dopamine release (Supplemental Fig. 4).

### 3.4 Response-cue contingency is necessary for elevated response rates under second-order schedule

**Fig. 6:**
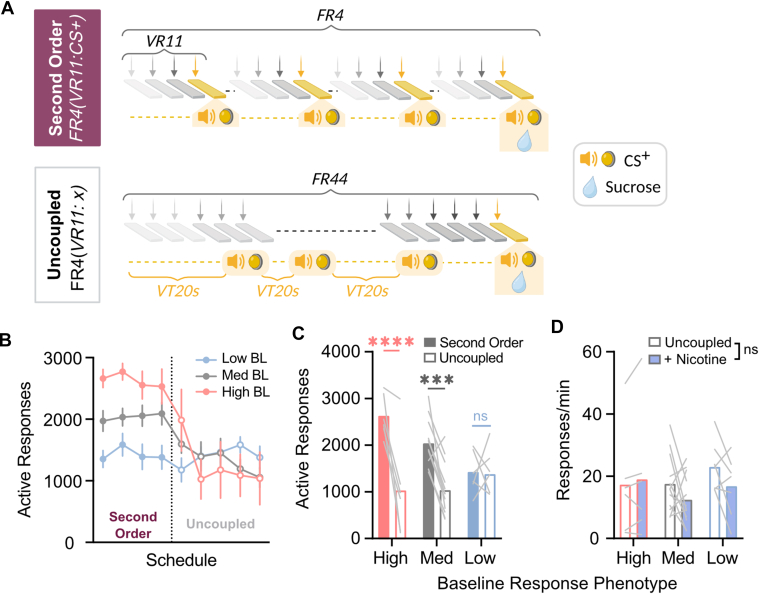
Uncoupling the response-CS+ contingency does not reduce operant responding for sucrose in low baseline responders. (A) Schematic of second-order procedure (top) and uncoupled procedure (bottom). In the uncoupled procedure additional CS+ presentations were interleaved on a variable time schedule, thus resulting in their presentation in a manner not directly linked to lever pressing. (B) Second-order schedule performance was compared between contingent CS+ presentation and noncontingent (uncoupled) CS+ presentation across low, medium, and high-baseline (BL) responder subgroups. (C) Uncoupling of the response-CS+ contingency decreased active responses in the high and medium baseline responders, but had no effect in the low baseline responder group. (D) Nicotine pretreatment did not influence response rates when the CS+-contingency was uncoupled from responding. Data are presented as mean ± SEM. ∗∗∗*P* < .001, ∗∗∗∗*P* < .0001, ns = not significant.

To assess the role of cue–response contingency, mice were tested under a schedule in which CS+ presentations were decoupled from lever pressing (Fig. 6A). Two-way ANOVA revealed a main effect of schedule (Fig. 6B; *F*_1,21_ = 31.35, *P* < .0001), as well as an interaction (schedule × phenotype: *F*_2,21_ = 7.363, *P* < .01). High and medium baseline responders displayed reduced lever-pressing after contingency degradation (Fig. 6C; High: Bonferroni *t* = 5.274, *P* < .0001; Med: Bonferroni *t* = 4.617, *P* < .001), whereas low baseline responders maintained stable response rates, regardless of cue contingency (*t* = 0.1132, n.s.). Although the procedure was not designed to prevent spontaneous coincidence between responses and noncontingent CS+ presentations (eg, change-over-delay^50^^,^^51^), we observed that mice with the highest rates of responding—and, thus, probabilistically most likely to emit a response around a scheduled CS+—exhibited the most robust reduction in behavior during contingency degradation (Fig. 6, B and C). Therefore, spontaneous response-cue pairings likely did not preserve the conditioned reinforcing effects of the CS+.

This suggests that conditioned reinforcement, as defined by response-dependent CS+ presentation, is not critical to operant performance in mice that exhibit lower response rates under a second-order schedule. Although response rates were comparable under both schedules, a dose of nicotine that stimulated responding in low baseline responders under the second-order schedule failed to augment responding when the CS+ was uncoupled from lever pressing (Fig. 6D; *F*_1,21_ = 2.218, n.s).

## 4 Discussion

Nicotine’s ability to dose-dependently enhance dopamine responses to conditioned cues in the DLS predicted the ability of nicotine to modulate cue-motivated behavior. Nicotine effects on behavior were complex and were influenced by a range of factors, such as (1) whether behavior was maintained by a primary reinforcer or a conditioned reinforcer, (2) baseline response rate, and (3) the pharmacological actions of nicotine at nicotinic receptors. When behavior was maintained by sucrose as a primary reinforcer, nicotine had no effect under any conditions on behavioral performance. However, when behavior was maintained under a second-order schedule, there was a relationship between baseline rate of behavior and effects of nicotine such that it enhanced response rates selectively when they were low at baseline. Thus, the rate-enhancing effects of nicotine were revealed only under a second-order schedule but were limited to a subset of animals. Critically, these rate-dependent behavioral and neural effects were reversed by the nicotinic antagonist, mecamylamine, showing that the effects are mediated by nicotinic receptors. Phenotypically, mice sensitive to the effects of nicotine tended to be insensitive to the conditioned reinforcers at baseline; thus, it is tempting to speculate that nicotine may not simply increase behavior generally, but potentially influences strategies that animals use to guide their behavior.

Consistent with previous findings,^10^^,^^13^^,^^52^ our data indicate that nicotine’s influence on operant behavior is most clearly expressed in the presence of CS+. When sucrose delivery was maintained through direct response–outcome contingencies (first-order schedule), nicotine administration had negligible effects across a broad dose range. In contrast, when behavior was maintained by conditioned stimuli under a second-order schedule, nicotine selectively enhanced responding, but only in animals with low baseline response rates. Rate dependency has been a central tenet of behavioral pharmacology for >50 years, referring to the principle that a drug’s effects on behavior are systematically related to baseline rates of responding.^53^^,^^54^ In general, drugs tend to increase responding most prominently when baseline rates of behavior are low or decrease rates to a greater extent when predrug rates are high.^48^ Baseline-dependent effects of nicotine have been well established across a range of behavioral and cognitive endpoints, yet the effects on operant performance have been mixed.^49^ While our data are consistent with the fundamental rate-dependency framework (ie, nicotine selectively enhanced responding in animals with low baseline rates), this relationship was specific to behavior weakly maintained by conditioned reinforcers (under the same schedule of reinforcement), suggesting that rate or schedule alone could not explain these phenomena in their entirety.

The interaction between rate and conditioned stimuli suggests that these complex effects could be explained by latent variables that cannot be captured simply by a unitary measure. For example, it is possible that low baseline responders implement different behavioral strategies: in this case, one that appears to discount the motivational relevance of conditioned cues. Indeed, low baseline responders were insensitive to the removal of the cue–response contingency and maintained similar rates of responding when the CS+ was uncoupled from behavior. Importantly, the enhancement of operant behavior by nicotine required intact action–outcome contingencies, as uncoupling cue presentation from lever-pressing eliminated nicotine’s effects. Thus, nicotine may enhance the likelihood that mice will use cues to guide their behavior by augmenting the salience of these cues. These findings align with some early observations that the behavioral relevance and salience of external stimuli are critical to explaining the relationship between baseline rate and drug effects under some conditions. For example, the rate-enhancing effects of amobarbital were found to be most pronounced when behavior was maintained by weak visual stimuli (ie, a dim cue light), and were entirely mitigated in the presence of more salient instructive cues.^55^ However, the introduction of task-relevant stimuli, such as a “clock” indicating time-passage under an interval schedule, can dramatically reduce the rate-enhancing effects of several drug classes.^56^ In both cases, drug effects were best predicted by an animal’s reliance on external cues for behavioral control rather than baseline response rates alone. Together with our data, these studies suggest that nicotine’s ability to modulate behavior partly reflects its capacity to augment the influence of external stimuli that guide action selection, particularly under conditions of weak baseline stimulus control. These findings further highlight how individual differences in baseline behavioral strategies critically shape susceptibility to nicotine’s reinforcing effects.

Nicotine’s enhancement of operant responding was paralleled by amplification of cue-evoked dopamine release in the DLS of low baseline responders, suggesting that rate dependency at the behavioral level reflects underlying differences in how nicotine engages reinforcement-related dopamine signaling. These rate-dependent effects were mediated by nicotinic acetylcholine receptors, as it was abolished by mecamylamine pretreatment. Furthermore, nicotine amplified cue-evoked dopamine release in the DLS without affecting dopamine dynamics in the NAc, suggesting projection- and phenotype-specific modulation of reinforcement processes. These data suggest that nicotine’s ability to enhance conditioned reinforcement is linked to potentiation of dopamine release specifically in striatal regions associated with stimulus–response learning and habitual behavior.^34^^,^^57^, ^58^, ^59^, ^60^ Indeed, it has been well established that drug-associated cues play a prominent role in perpetuating nicotine use dependence^61^, ^62^, ^63^; moreover, nicotine has been shown to generally augment dorsal striatal response to reward-related cues in humans.^64^, ^65^, ^66^ These findings are consistent with the idea that nicotinic modulation of DLS dopamine signaling may specifically permit reward-related cues in the environment to acquire heighted salience.^67^^,^^68^ While nicotine’s ability to amplify conditioned reinforcement likely generalizes to cues associated with a wide range of reinforcers, its effects may be most problematic in the context of drug reinforcement, in which the potentiation of cue-driven behavior could exacerbate drug seeking and relapse vulnerability. Indeed, nicotine use is associated with higher relapse rates than other drugs of abuse, underscoring the clinical relevance of these mechanisms.^69^ Together, these findings indicate that nicotine does not uniformly amplify striatal dopamine responses but rather selectively enhances dopamine signaling in circuits supporting cue-driven behavior in individuals with behavioral profiles that predict greater vulnerability to conditioned reinforcement.

Together, these findings extend classical concepts of rate dependency^48^^,^^70^^,^^71^ to the domain of conditioned reinforcement and identify a striatal circuit mechanism that may underlie individual susceptibility to nicotine’s behavioral effects. Furthermore, the data strengthen the idea that behavioral effects of nicotine are not solely determined by its pharmacological actions but emerge through dynamic interactions with environmental contingencies.^72^ The projection-specific modulation of dopamine signaling observed here further suggests that different striatal circuits are differentially recruited depending on the behavioral context and reinforcement history. Understanding how drugs like nicotine exploit cue-driven behavior at both the circuit and behavioral level provides critical insight into the mechanisms underlying persistent drug use and offers potential targets for interventions aimed at disrupting maladaptive reinforcement processes.

## Conflict of interest

The authors declare no conflicts of interest.
