# Supplementary material for: Nicotine enhances the ability of cues to control behavior and evoke dopamine release in the dorsolateral striatum
Source: J Pharmacol Exp Ther. 2025 Jul 22;392(9):103662. doi: 10.1016/j.jpet.2025.103662 (PMC12597647; doi:10.1016/j.jpet.2025.103662)
Supplement: Supplementary Figures 1-4 [file mmc1.pdf]

Supplementary Materials

Journal of Pharmacology and Experimental Therapeutics

Nicotine enhances the ability of cues to control behavior and evoke dopamine release in the dorsolateral striatum

Michael Z. Leonard[1,2#], Hannah B. Elam[1,2#], Hye Jean Yoon[1,2#], Sofia H. Lago[1], Megan E. Altemus[1,2], Shemuel Roberts[1,2], Maxime Chevée[1,2], Erin S. Calipari*[1,2]
[1] Department of Pharmacology, Vanderbilt University, Nashville, TN
[2] Vanderbilt Center for Addiction Research, Vanderbilt University, Nashville, TN, 37232, USA
#Authors contributed equally

* Corresponding Author
Erin S. Calipari
<u>Director</u>
Vanderbilt Center for Addiction Research
<u>Associate Professor</u>
Department of Pharmacology
Vanderbilt University
2220 Pierce Avenue, Nashville, TN 37232
erin.calipari@vanderbilt.edu

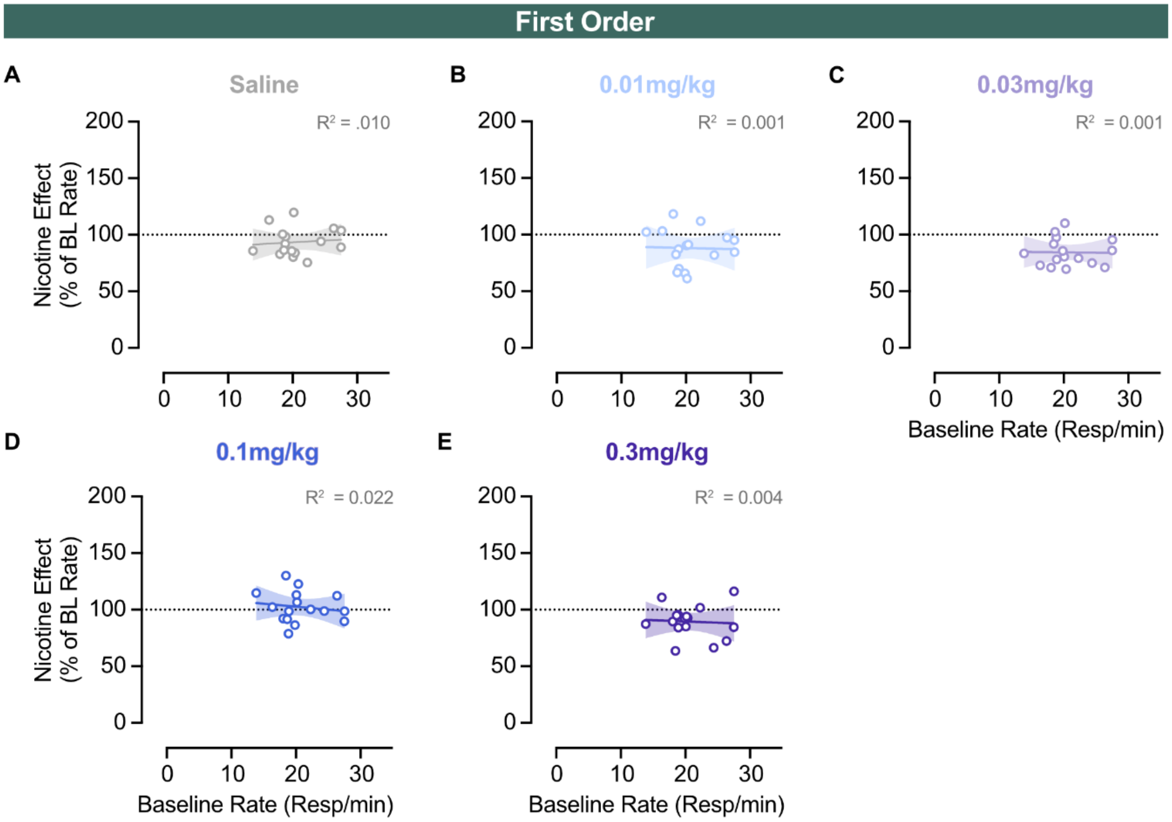

Supplemental Figure 1. Rate-dependent effects on sucrose responding were not observed under a first-order VR11 schedule across nicotine doses. Linear regressions comparing baseline responding with response-rate following **A)** saline **B)** 0.01mg/kg nicotine **C)** 0.03mg/kg nicotine **D)** 0.1mg/kg nicotine **E)** 0.3mg/kg nicotine.

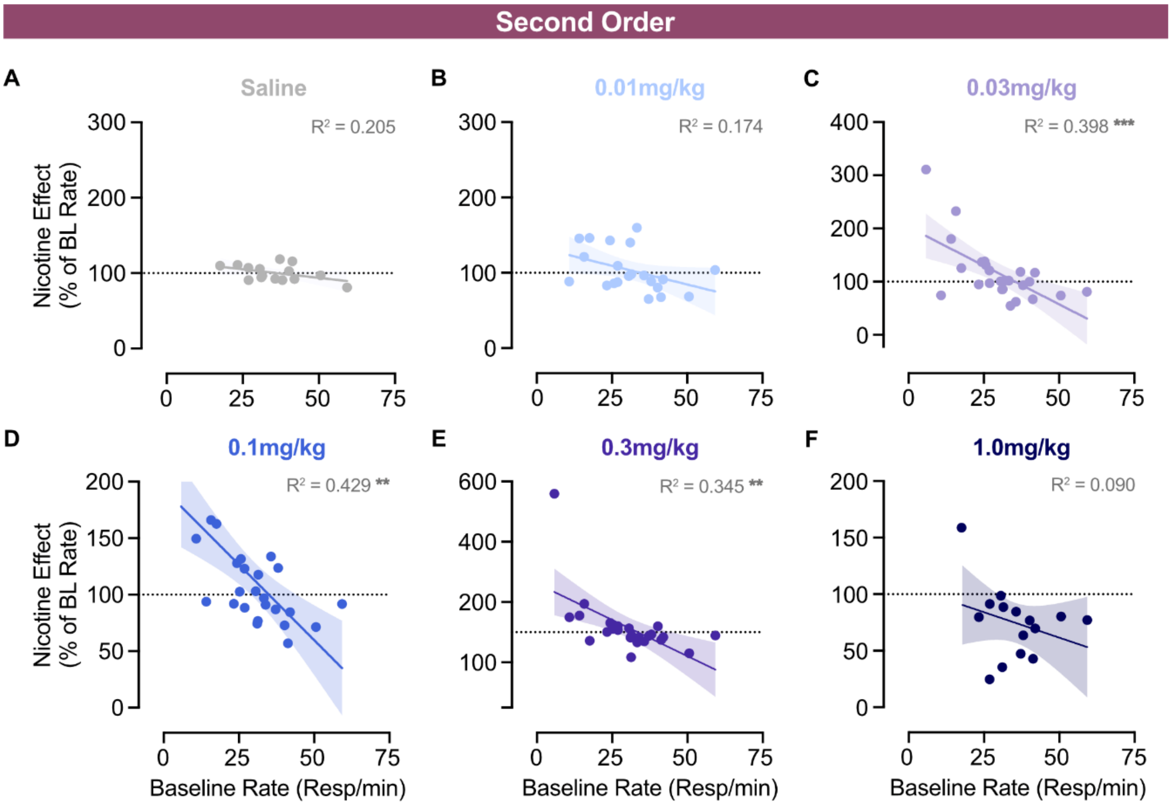

Supplemental Figure 2. Nicotine's rate-dependent effects on sucrose responding were observed under second-order schedules in a dose-dependent manner. Response rate was unchanged following **A)** saline and **B)** 0.01mg/kg nicotine. A relationship between baseline response rate and nicotine-induced responding was revealed after **C)** 0.03mg/kg nicotine **D)** 0.1mg/kg nicotine and **E)** 0.3mg/kg nicotine. **F)** Responding after 1.0 mg/kg of nicotine had no relationship with baseline response-rate. Data are presented as mean ± SEM. ** $p<0.01$, *** $p<0.001$.

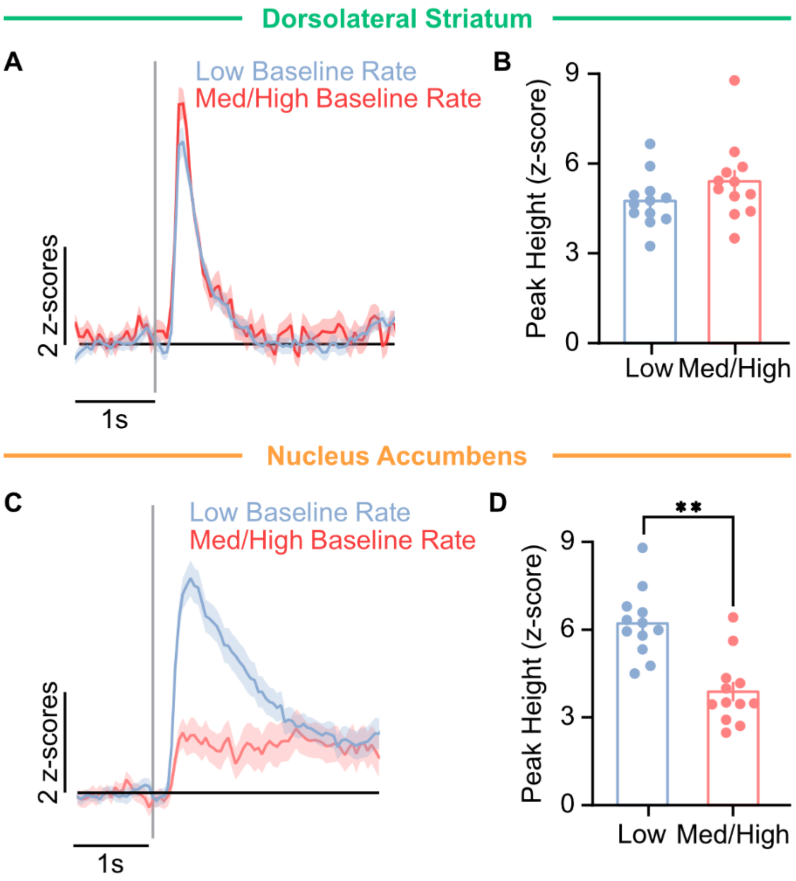

Supplemental Figure 3. CS+-evoked dopamine responses in the NAc differ at baseline between high and low responders. A) Baseline CS+-evoked dLight1.1 photometry traces in the DLS from med/high and low responders. **B)** Peak dopamine responses did not differ between the two groups at baseline. **C)** Baseline CS+-evoked dLight1.1 photometry traces in the NAc. **D)** Peak dopamine responses were greater at baseline between high and low responders. Data are presented as mean ± SEM. ** $p<0.01$.

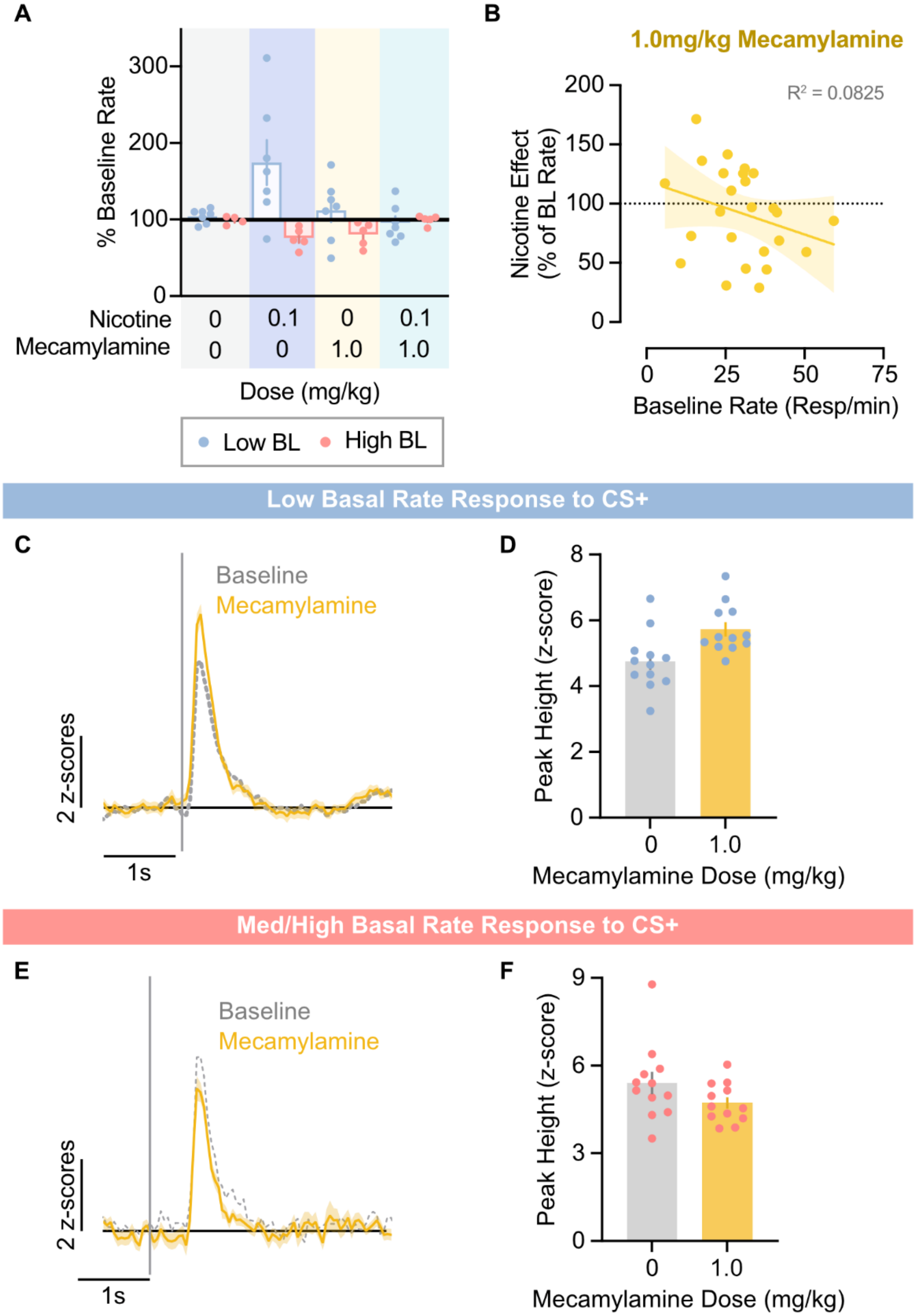
A
% Baseline Rate
300
200
100
0
Nicotine 0 0.1 0 0.1
Mecamylamine 0 0 1.0 1.0
Dose (mg/kg)
Low BL
High BL
B
1.0mg/kg Mecamylamine
$R^2 = 0.0825$
Nicotine Effect (% of BL Rate)
200
150
100
50
0
0 25 50 75
Baseline Rate (Resp/min)
Low Basal Rate Response to CS+
C
Baseline
Mecamylamine
2 z-scores
1s
D
Peak Height (z-score)
8
6
4
2
0
0 1.0
Mecamylamine Dose (mg/kg)
Med/High Basal Rate Response to CS+
E
Baseline
Mecamylamine
2 z-scores
1s
F
Peak Height (z-score)
9
6
3
0
0 1.0
Mecamylamine Dose (mg/kg)

Supplemental Figure 4. Mecamylamine alone had no effects on response rates or dopamine dynamics. A) Response rates, separated by phenotype, following nicotine alone, mecamylamine alone, and a combination of nicotine and mecamylamine. **B)** No relationship between baseline response rate and response rate following mecamylamine. dLight photometry traces from the DLS time-locked to CS+ (grey line) presentation in **C)** low baseline and **E)** medium and high baseline responders, following mecamylamine (1.0mg/kg, i.p.). Mecamylamine had no effect on peak dopamine responses in **D)** low baseline responders or **F)** med/high baseline responders.
